# From climate scepticism to discourses of delay in UK editorials

**DOI:** 10.1177/09636625251315446

**Published:** 2025-03-11

**Authors:** Sylvia Hayes, Josh Gabbatiss, Catherine Butler

**Affiliations:** University of Exeter, UK; Carbon Brief, UK; University of Exeter, UK

**Keywords:** climate action, climate change communication, energy supply, opinion journalism

## Abstract

News media has long been recognised for its important role in shaping public discourse and socio-political action relating to climate change. This is particularly true of opinion journalism, which reflects elite voices. Within the climate change communications literature, an important shift marks a turn away from outright denial of the existence of climate change towards delaying narratives. In this paper, we use a longitudinal mixed-methods analysis to chart ‘discourses of delay’ in editorials relating not only directly to climate change but to the closely connected issue of energy transitions across seventeen UK daily and weekly newspapers over the period 2011–2021. Though we find both a trend away from outright denial of climate change and an identifiable increase in support for climate action even among right-leaning editorials over this period, we also show that narratives are characterised by multiple discourses of delay across both climate change and various energy narratives.

## 1. Introduction

News media can have a significant impact on people’s understanding of an issue as complex as climate change, and the body of research investigating the communication of climate change in the media has become substantial enough to be deemed a field of research in its own right (Moser, 2016; Pearce et al., 2015). This vast body of research has suggested that print media narratives often misrepresent climate science messages and are highly influenced by ideological or socio-political context (Boykoff, 2011; Broadbent et al., 2016). For example, the political orientation of a newspaper has been found to have an influence on the way that climate change is framed in news content (Carvalho, 2007; Carvalho and Burgess, 2005). To date, however, the focus of analysis has been on news articles and the research on UK editorial and/or opinion journalism is ‘scant’ (Painter and Gavin, 2016: 437). This is despite the significance of these articles in both informing readers on complicated issues and representing the ‘broader ideological stance’ of newspaper ownership and management (Henry and Tator, 2002; Painter and Gavin, 2016: 93). Though there is existing research investigating opinion journalism in the United States (e.g. see Golan and Lukito, 2017; Izadi and Saghaye-Biria, 2007; Richardson and Lancendorfer, 2004), there is little examination of climate change coverage and the UK context. This is an increasing concern for climate communication, given the way that issues of climate and energy have become co-opted as part of a wider culture war in UK politics. Debates around Net Zero and the Ultra Low Emissions Zone in London did become one of the most contested policies in recent UK politics, provoking intense scrutiny and debate from some media outlets and (from 2022), much newspaper column space given to criticising related policies including on Low Traffic Neighbourhoods (Evans, 2023), fracking (Daley et al., 2024; Paterson et al., 2023) and the economic cost of Net Zero policies (Ward, 2023).

Editorials operate differently to general news reporting in multiple ways. First, editorials do not cater predominantly to general public audiences, instead reflecting the issues and opinions of elite players (Henry and Tator, 2002). Second, McChesney (2004) describes the lack of standard journalistic norms of balance and objectivity associated with editorials, resulting in content that contains political opinions. Editorials thus present an important area of study to not only more fully understand media discourses surrounding climate change action, but also to understand the position of elite players in society in relation to these issues. Given that climate change action requires significant changes from elite actors specifically (Cambridge Sustainability Commission, 2021), this marks such journalistic forms out as particularly important. Similarly, Painter and Gavin (2016) found that the majority of articles containing climate sceptical voices were published as opinion pieces from only two of the seventeen newspapers studied (The Telegraph and The Express), indicating the significance of opinion journalism as a potential site of anti-climate action sentiment. There is therefore a need to investigate the role of opinion journalism in relation to climate change scepticism.

Recent developments in academic research have focused on the emergence of a different tone of climate change scepticism, which has been termed ‘discourses of delay’ (Coan et al., 2021; Lamb et al., 2020: 1). This refers to discourses and narratives which ‘accept the existence of climate change, but justify inaction or inadequate efforts’ (Lamb et al., 2020: 1). The literature on ‘delay’ discourses is emergent, but shows the importance of understanding this shift across areas in the UK context such as public attitudes towards lifestyle change (Cherry et al., 2024) and in political debate (Nisbett et al., 2024). We build on this important work by applying these concepts to editorials in UK newspapers. Indeed, despite the substantial body of literature on media coverage of climate change, Boykoff et al. (2021) point to persisting research gaps, particularly around opinion journalism (as opposed to news stories) and newer forms of discourse including ‘discourses of delay’. It is these gaps which the current study aims to address by taking forward analysis in two key ways.

First, we bring focus on contemporary editorials by applying a mixed-methods longitudinal analysis to over 1000 such pieces published across 17 UK newspapers from the period 2011–2021. Second, we extend the analysis beyond a straightforward focus on climate change scepticism and opposition to look at articles relating to forms of energy supply that are directly implicated in debates about climate action. In this our analysis spans renewable energy (predominantly wind power), nuclear power and gas fracking. These three sources of energy supply were the most commonly discussed forms across our data set and all are integral to debates about action on climate change. Though all these forms of energy supply have been the focus of media analysis separately (e.g. Culley et al., 2010; Djerf-Pierre et al., 2015; Philo and Happer, 2013), there has been little work drawing together analysis of climate change scepticism with that of energy systems and their transition to address climate change. This is important not only because these concerns are intimately interconnected but because the shifts in analysis of climate change scepticism bring focus on the wider narratives that belie inaction.

Through the analysis, then, the paper aims to reveal both the extent and nature of both pro- and anti-climate action stances across both right- and left-leaning UK newspaper editorials and the ways that discourses about energy supply can feed into this. We show that though outright denial of the existence and severity of climate change have declined across the board, other narratives downplaying the need for action on climate change remain. We identify multiple delay narratives relating to redirecting responsibility and pushing non-transformative solutions, emphasising the downsides of action and the delegitimisation of those who support action. We offer, therefore, a contribution to understanding such forms of opposition by showing how delay narratives emerge both within editorials about climate change and, more subtly, within pieces relating to energy supply and transitions.

## 2. Advances in understanding media representations of climate change scepticism

There are continued debates about defining and differentiating between different types of climate change scepticism. Entering into these debates is beyond the scope of this article, and instead this research adopts the taxonomy of scepticism formalised by Painter and Ashe (2012: 3), which identifies three types of sceptics: (1) ‘trend sceptics’, who deny the warming trend; (2) ‘attribution sceptics’, who question the anthropogenic nature of the trend and (3) ‘impact sceptics’, who accept anthropogenic warming but question the need for policy intervention, claiming impacts may be positive or negligible.

One of the most commonly studied areas of climate change news coverage regards the way that a climate change counter-movement has influenced media coverage with a sceptical or denial focus. This movement was made up of conservative thinkers, foundations, journalists and politicians and aimed primarily to influence media coverage about climate change in order to instil doubt around the scientific evidence for climate change (Fahy, 2017). Though a majority of studies focus on the US context, recent research has shown that this countermovement is international and particularly present in the United Kingdom (Almiron et al., 2023). Jacques et al. (2008) found that over 92% of English-language climate sceptical books published between 1975 and 2005 were linked to Conservative Think Tanks. In an analysis of the counterclaims published in official documents of the organisations which make up the climate change counter-movement, McCright and Dunlap (2000) found three broad claims dominated: (1) ‘the evidence for global warming is weak or wrong’; (2) ‘global warming will be beneficial if it occurs’; (3) ‘policies to address global warming would be more harmful than helpful’ (p. 780). As demonstrated by these various taxonomies, the pervasiveness of climate change scepticism goes beyond simply denying or questioning the scientific evidence for warming.

A more recent body of work has addressed this terminological issue, with many discussing the difficulties with labelling all narratives which do not support climate change action as ‘denial’. Multiple authors have called for a reconsideration of this term and a broader discussion of the ways in which climate change action is stymied or opposed in the contemporary media landscape (Almiron and Moreno, 2022; Lamb et al., 2020; McMie, 2021). Almiron and Moreno (2022) discuss how the majority of ‘dissidents’ in fact do not deny the existence of global warming but instead are opposed to the political changes and policies that seek to address the problem. Instead, the authors put forward the terms ‘delay’, ‘opposition’, ‘contrarianism’ and ‘obstructionism’ as better suited to reflect the complex nature of opposition to action on climate change. Almiron and Moreno (2022) argue that the majority group of ‘dissents’ are ‘obstructionists’.

The term ‘obstructionism’ in this context specifically refers to messaging which aims to delay or obstruct climate action and represents the ‘evolving misinformation playbook’ of the climate change counter-movement (Holder et al., 2023: 1). Obstructionism in the literature acknowledges the intentions of the organised campaign to push against climate change action (Dunlap and Brulle, 2020) in line with the interests of the groups in the climate change counter-movement (Almiron and Moreno, 2022; Dunlap and Brulle, 2020). The term ‘obstructionism’ therefore speaks to how the reduction of arguments to denier/non-denier makes invisible the ideology of patriarchal-industrial capitalism which actually underpins much of the obstructionist arguments (Almiron and Moreno, 2022). The emerging literature on discourses of delay has shown that obstructionist arguments are in use on Facebook advertisements from fossil fuel companies, advocacy organisations and industry associations (Holder et al., 2023).

It is from this context that the term ‘discourses of delay’ emerged in the literature. The idea of discourses of delay builds on the concept of obstructionism and relates to the narratives and messages which do not deny the existence of climate change, but aim to *delay* policies or actions which would mitigate the impacts of it. A widely cited paper on discourses of delay from Lamb et al. (2020: 1) defines discourses of delay as ‘policy-focused discourses that exploit contemporary discussions on what action should be taken, how fast, who bears responsibility and where costs and benefits should be allocated’. According to Lamb et al. (2020), there are four groups of delay discourses, (1) redirect responsibility, arguing that ‘someone else’ should take action first; (2) push non-transformative solutions, arguing that disruptive and substantial societal change is not required; (3) emphasise the downsides of climate policies, e.g. focusing on the economic cost of renewable energy and (4) surrender to climate change, which refers to the narrative of doomism that it is too late for mitigation.

This typology has emerged developed from the ‘collective observations’ of social scientists studying climate change (Lamb et al., 2020: 1) and, to date, has not been subject to much empirical examination (with exceptions, see Coan et al., 2021), despite key advances in the literature around the difference between denial and delay tactics (e.g. Cherry et al., 2024; Nisbett et al., 2024). This article offers a key contribution to understanding discourses of delay by analysing UK news editorial articles to better understand how such narratives arise and are articulated in diverse ways within a key form of public discourse.

Within media research, few studies have examined media coverage of issues such as energy transitions which are related to climate change coverage. Understanding the media coverage of these related issues would help us better understand how such delay or oppositional narratives manifest and operate outside of overtly denialist climate change discourse. Energy supply, in particular, seems important to study given the ways that energy issues have become an increasingly important global topic, gaining widespread attention in the British media amid the ongoing ‘energy crisis’ (Philo and Happer, 2013). It is in this context that the present research sits, analysing not only climate change editorials but also those discussing various energy transition issues.

Energy has come to be frequently addressed in multiple ways across news media including in relation to energy transitions, energy efficiency, controversies and debates about different forms of energy supply, and energy companies and markets. While it is likely to be important to examine energy discourse more widely within climate change communication research, here we focus on articles relating to three forms of energy supply that were dominant across our data set and are highly pertinent to debates about climate change action within the United Kingdom – renewables, nuclear and fracking. There is an existing body of media research on stories about energy sources and forms of supply (e.g. see Philo and Happer, 2013 for an overview). However, the step to examine articles about energy supply in the context of climate change scepticism narratives and discourses of delay has yet to be taken.

This study provides a longitudinal analysis of UK print media editorial coverage of climate change and energy supply issues over the period 2011–2021. In light of the literature, we posed the following research questions for the analysis:

1. How frequently have UK newspaper editorials represented (1) a pro-climate action stance or (2) an anti-climate action stance, over the period 2011–2021?
2. What evidence is there of *discourses of delay* in UK newspaper editorials between 2011 and 2021 when discussing climate change and three energy sources?
3. How do discourses of delay emerge within UK newspaper editorials and what narrative forms do they take across politically left and right-wing sources?

## 3. Research design and methods

This research uses a longitudinal mixed method analytic approach, including quantitative content analysis and qualitative discourse analysis, to investigate changes in UK national newspaper editorial discourses on climate change action and energy sources over the period 2011–2021 and across publications with different political orientations.

### Data collection

Editorials (total *n* = 1342) were collected from 17 UK newspapers, including a range of daily and Sunday papers, and from a range of ideological or political positions, both broadsheet and tabloids. Data collection was manual, using keywords and a mixture of RSS feeds and Factiva. Two datasets were created: one containing climate change editorials and one containing energy editorials. The climate dataset is made up of editorials containing one or more of the keywords: ‘climate change’ or ‘climate action’ or ‘global warming’ or ‘global heating’ or ‘greenhouse gas emissions’ or ‘Paris Agreement’ or ‘Paris climate deal’ or ‘COP26’. The energy dataset is made up of editorials containing one or more of the keywords: ‘wind power’ or ‘wind farm’ or ‘wind energy’ or ‘renewable’ or ‘nuclear power’ or ‘nuclear energy’ or ‘fracking’ or ‘shale gas’ or ‘renewable energy’ or ‘renewable power’ or ‘solar power’ or ‘solar energy’ or ‘solar’ or ‘hydraulic fracturing’. The energy sources of nuclear power, fracking and renewables were chosen as these were found to be the most common in the dataset through piloting. Newspapers and keywords were selected according to expertise of the authors and building on previous similar studies. Although the issues of climate change and energy supply are interconnected in this context, the two datasets were treated separately because piloting revealed that editorials rarely discussed the two together; when energy supply sources were discussed, they were very rarely linked to the wider issue of climate change. The climate dataset contained 712 editorials which were coded, and the energy dataset contained 507 which were coded. Of these, 201 editorials appeared in both datasets.

Table 1 shows the number of editorials coded from each newspaper in each dataset and the ideology assigned to each according to a YouGov poll (Smith, 2017). In Table 1, ‘L’ refers to a left-leaning political ideology, ‘R’ refers to a right-leaning, and ‘C’ refers to a centre.

**Table 1.** Number of editorials from each publication which made up each of the climate and energy datasets. Note that this contains only those editorials which were able to be coded, not all those which were collected. For details on exclusion/inclusion criteria, please see Supplemental Information 2: codebook.

Publication	CLIMATE DATASET	ENERGY DATASET	Categorised newspaper ideology
	Number of editorials coded	Number of editorials coded	
*The Guardian*	189	77	L
*Financial Times*	143	93	C
*The Times*	75	82	R
*The Independent*	65	43	C
*The Daily Telegraph*	49	53	R
*The Sun*	51	53	R
*The Daily Mail*	43	30	R
*The Observer*	29	15	L
*The Daily Mirror*	26	9	L
*Daily Express*	19	18	R
*The Sunday Times*	11	7	R
*The Mail on Sunday*	2	6	R
*The Independent on Sunday*	2	3	C
*Sunday Express*	1	6	R
*The Sun on Sunday*	3	3	R
*The Sunday Telegraph*	3	9	R
*The Sunday Mirror*	1	0	L
*Total*	*712*	*507*	

### Analysis

Editorials were analysed using a mixed-methods approach of quantitative content analysis and qualitative discourse analysis.

The quantitative analysis captured the overall position of the editorial (e.g. does the editorial overall call for more stringent climate action policies?) and also themes present in the text (e.g. does the editorial emphasise the economic benefits of action?). First, all editorials were read in full and assigned a code according to whether the overall stance of the editorial broadly called for ‘more climate action’ or ‘less or no action’, which was defined as ‘Editorial calls for either *no* action to mitigate the effects of climate change, OR calls for *less* action than is currently in place’. The third category was ‘mixed/unclear’, for editorials which were purely informational (e.g. explaining the science of climate change or explaining a specific event without taking a position on whether action should be taken or not), or arguing contradictory points. Second, editorials were analysed for the themes present, with separate themes present in the climate and energy datasets. Separate codebooks were used for each of the climate and energy datasets. See Supplemental Information for both full codebooks.

Both codebooks were developed iteratively, and two authors were involved in the process. Provisional codebooks were created using a mixture of inductive and deductive methods (Matthes and Kohring, 2008) inspired by previous media analyses (see, e.g., Culley et al., 2010; Djerf-Pierre et al., 2015; O’Neill et al., 2015; Rochyadi-Reetz et al., 2019). For example, the code ‘economic cost’ when discussing an energy source was developed from literature examples (e.g. Culley et al., 2010; Delshad and Raymond, 2013), whereas the code ‘energy demand’ as justification for an energy source was developed inductively from the dataset.

To ensure codebook rigour, both codebooks were tested and piloted. Two authors then independently coded the same 10% sample of articles from both datasets. Average percentage agreement from this comparison was 92%, and the mean Krippendorf’s Alpha score of inter-coder reliability was 0.72. This inter-coder reliability was treated as an initial point to refine the codebook, and both authors met to discuss all disagreements in the coding, each explaining possible reasons for differences and with an eventual consensus reached on each code. The initial codebooks were then updated and edited to reflect any changes that emerged from this discussion. Coding was then undertaken by a single coder, given the level of agreement and collaboratively designed codebooks.

Following this, a qualitative discourse analysis approach was taken, to understand the relationship between themes present in the dataset and the discourses outlined in Lamb et al.’s typology. This enabled direct links with the typology to be revealed not only to address the research questions but also to reveal nuances and other distinctive delay narratives. Conducting the analysis in this way allowed for inductive themes to emerge from the dataset rather than relying solely on the a priori typology of Lamb et al. (2020).

A simplified version of each codebook is provided below, in Table 2 (climate) and Table 3 (energy), including a short description of each theme, along with the discourse of delay which they relate to.

**Table 2.** Simplified codebook for climate dataset, including the relationship between themes arguing for less action and the discourses of delay from Lamb et al. (2020). See supplemental information for full codebook. Note that grey highlighted themes argue for ‘less/no climate action’ and the white themes argue for ‘more climate action’.

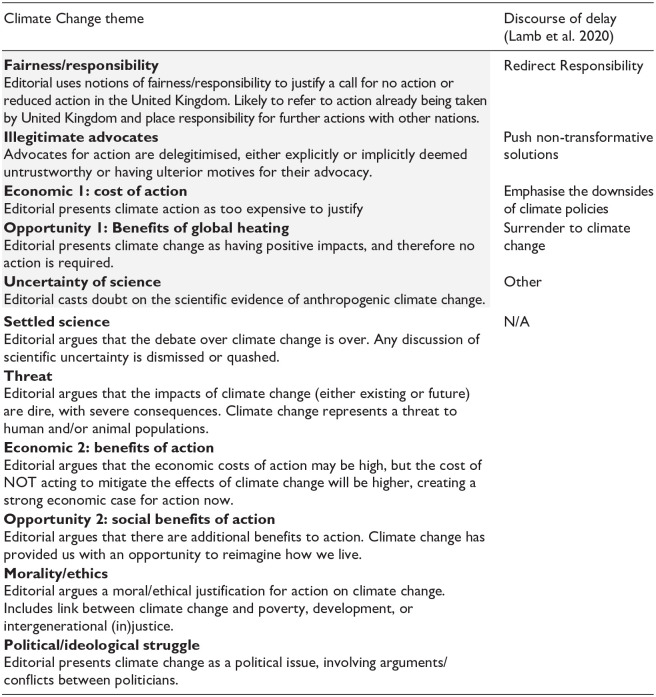

**Table 3.** Simplified codebook for energy dataset. See supplemental information for full codebook. Note that grey highlighted themes argue against the use of the energy source, and the white themes argue for the use of the energy source.

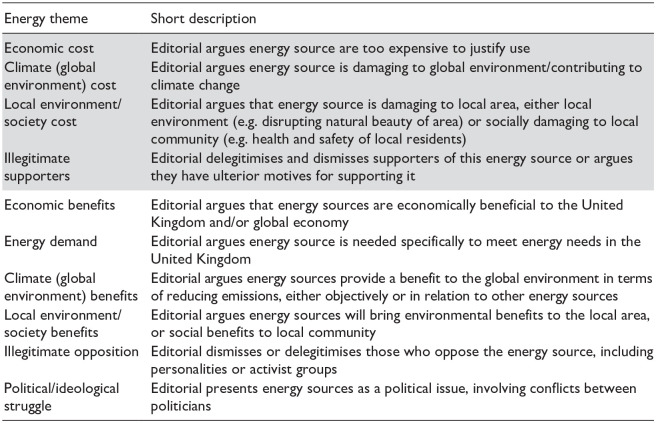

For the energy dataset, themes mapped onto the discourses of delay typology in a more complex way, as the themes work across different energy supply options (fracking, renewables and nuclear). For example, our ‘economic cost’ code (arguing that an energy source is too expensive to justify use) could be seen as a delay discourse if about renewable energy, but the code could act as a pro-climate action discourse when used to discuss gas fracking instead. This complexity meant we have not mapped across from our themes to Lamb et al.’s (2020) discourses of delay for the energy dataset in Table 3 as we have done for the climate change dataset in Table 2.

## 4. Discourses of climate scepticism and delay in UK editorials

The analysis offers important insights into both how climate change and energy editorial reporting have shifted over time in ways that highlight the relevance of discourses of delay to understanding contemporary climate scepticism. In what follows, we reflect on the outcomes of the analysis that shows how support for climate change in UK newspaper editorials across different political orientations has altered over time, with a significant decline in direct scepticism about both climate change science and the need for action. We then move to discuss the undercurrent of narratives which can be characterised in terms of Lamb et al.’s (2020) ‘discourses of delay’, present across both the climate and energy datasets.

### Overt climate change scepticism and its decline

**Figure 1.**
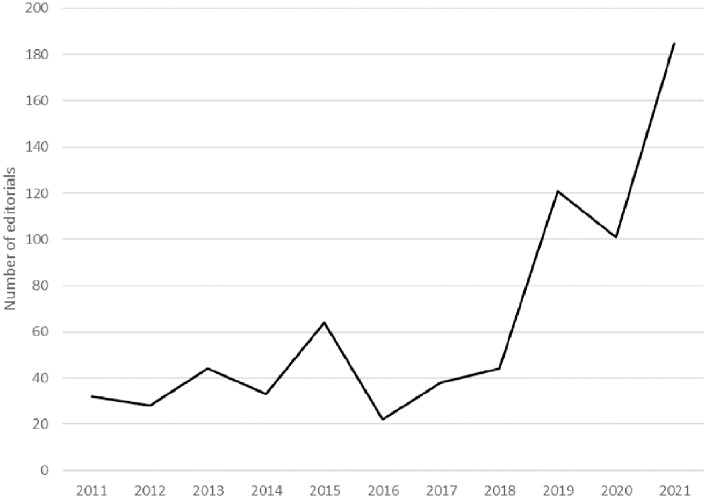
Number of newspaper editorials coded in the climate dataset each year.

To begin with our analysis of overt climate change scepticism, Figure 1 shows that the number of editorials discussing climate change remained low between 2011 and 2018, with a notable peak in 2015 (likely due to the Paris Agreement of 2015). In 2019, the number of editorials discussing climate change increased dramatically to nearly three times the number published in 2018. More than half the total coded editorials published in the 10-year period 2011–2021 were published in the last 3 years of the period. As shown below, this increase can be attributed almost exclusively to editorials calling for more action to address climate change and reflects the impact of national and global events in that period, such as widespread climate protests (Marris, 2019), the announcement of the UK’s net-zero target, and the COP26 climate change summit in Glasgow, all of which put climate change firmly on the UK’s news media agenda.

Perhaps more importantly than the level of coverage, the analysis shows how those articles were characterised by narratives that confirmed the scientific consensus about climate change. Headlines and text from traditionally more sceptical publications such as the Times took a notable turn towards reflecting the scientific consensus in their discourse: ‘Climate change is real and humans are the main cause’ (The Times, 28/09/2013), or later in 2018 ‘There is little doubt that the increased occurrence of extreme weather can be attributed to global warming. Climatologists believe that what had been “once in a century” events are likely to become “once in a decade” or less in many cases’ (The Times, 04/01/2018).

**Figure 2.**
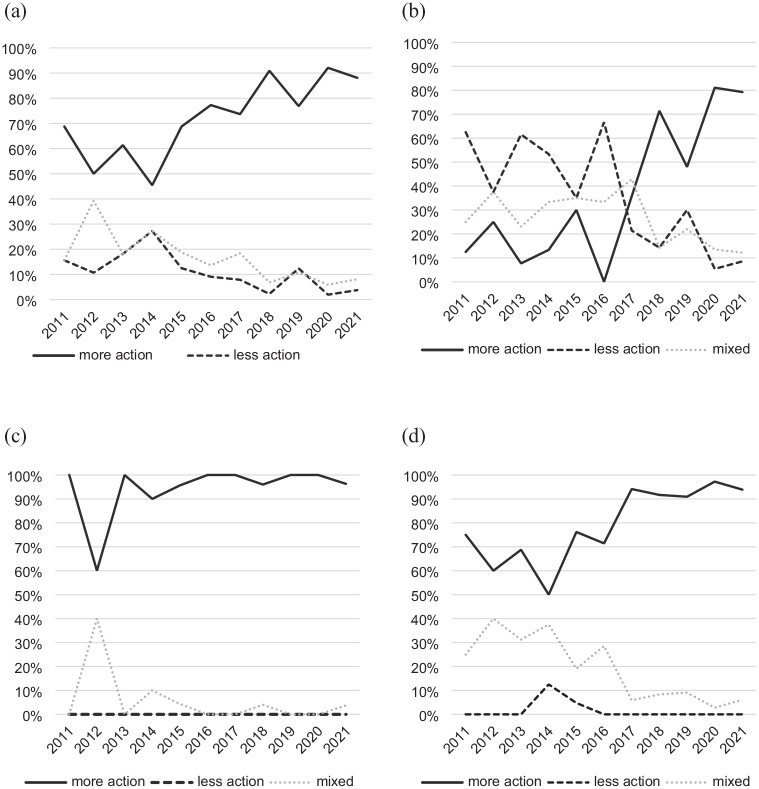
a, b, c, d: Editorials each year coded as ‘more action’, ‘less action’ or ‘mixed’, shown as percentage of total editorials coded each year for each ideology: (a) all publications, (b) right-leaning, (c) left-leaning and (d) centre.

Significantly, when thinking about discourses of delay, the analysis also shows how support for climate action within these editorials shifted over this period. Figure 2a shows that between 2011 and 2021, there were consistently more editorials that support climate action than those that oppose it.

This narrative turn within the climate change editorials was evident across the data set, indicating the dominance of discourse in UK newspaper editorials that emphasises the need for action to mitigate climate change. Figure 2a shows a clear upwards trend in the proportion of editorials supporting climate action from 2014 (where pro-climate action editorials made up less than half of editorials that year) to 2021 (where pro-climate action editorials represented 88% of editorials that year). This suggests that narratives of climate action have become a mainstream position for UK newspaper editorials in recent years.

This is perhaps reflective of a desire to resonate with audiences amid increasing proportions (45%) of the UK public being found to be ‘very’ or ‘extremely’ worried about climate change, and the recognition that climate change has become a permanent member of the UK public’s ‘pool of worry’ (CAST Centre, 2021; Evensen et al., 2021: 2). In terms of the nature of the discourse within these wider trends, the most common narrative themes relating to climate action in the dataset were ‘threat’ (present in 31% of climate editorials coded), ‘morality/ethics’ (21%), and ‘economic benefits’ (19%). Example quotes from editorials in the climate dataset which demonstrate each of these themes are presented below.

**Threat:** ‘The climate crisis is then a different order of challenge to anything seen in recent decades – a relentless, formidable, and existential threat’. (The Independent, 09/12/2020)

**Morality/ethics:** ‘Today’s leaders have a shot at preventing disasters which could define the lives of our grandchildren. They must not duck this’. (The Daily Express, 06/08/2021)

**Economic benefits (of action):** ‘While the bill for tackling climate change is unquestionably high, the bill for doing nothing is far higher’. (The Times, 31/07/2021)

Interestingly, it is the position of the right-leaning publications that showed the most striking shift in trends, as shown in Figure 2b. In 2011, only 13% of right-leaning editorials supported climate action and 63% opposed it, but by 2021, 79% supported climate action and only 9% opposed it. 2017 marked the first year that right-leaning publications published more editorials supporting climate action than opposing it, and more than 90% of the pro-climate action editorials from right-leaning publications were published in 2017 or later.

Previous studies have found right-leaning publications are less likely than left-leaning to support climate action (Painter, 2013). However, this analysis suggests that the ideological divide in support for climate action appears not as clear cut as it once was, with right-leaning publications almost exclusively publishing editorials that represent a pro-climate action stance in recent years, for example, ‘Even if Glasgow does not achieve its aims, the climate message is getting through and there is momentum for concerted action. And that is the most important thing’ (The Times, 31/10/2021, *climate dataset*).

The results show that the ‘scientific uncertainty’ code has essentially vanished from UK newspaper editorials over the period 2011–2021. While this code was always only in a small subset of editorials (just 28 editorials in the whole climate dataset), in 2011 it was the most common of the themes justifying reduced climate action, being present in 15% of all coded editorials that year. From 2016 onwards, the uncertainty theme was present in an average of only 1% of editorials per year, and no editorial has included the ‘scientific uncertainty’ code since a Daily Telegraph article in 2018. The scientific uncertainty theme incorporates ideas from both Type 1 scepticism and Type 2 scepticism, and so findings echo those of recent studies showing the significant decline of these types of scepticism from media coverage (Boykoff et al., 2021). Findings show, therefore, that editorials in the United Kingdom largely stopped publishing overt climate change scepticism relating to the underlying science in the later part of the 2010s.

This is a positive story, then, for climate action and support, perhaps reflecting an increasing cross-party consensus on climate change in recent years and efforts by many to highlight both the severity and urgency of the issues. However, in a context of literature that highlights shifts from outright climate scepticism to ‘discourses of delay’ (Lamb et al., 2020), it is important to examine these more subtle but arguably equally problematic narratives. As a result, we discuss below the way that editorials reflected and reproduced discourses of delay from Lamb et al.’s typology.

### Discourses of delay in climate change and energy editorials

Within the editorials, we found there were forms of discourse across both the climate and energy datasets that could be characterised in terms of delay and as aligning in different ways with Lamb et al.’s (2020) original typology, as illustrated in Table 4.

**Table 4.** Simplified codebook for climate and energy datasets with the relationship between themes/codes and the discourses of delay from Lamb et al. (2020). See supplemental information for full codebooks.

Discourse of delay (Lamb et al. 2020)	Climate change dataset themes	Energy dataset themes
Redirect Responsibility	**Fairness** Editorial uses notions of fairness/responsibility to justify a call for no action or reduced action in the United Kingdom. Likely to refer to action already being taken by the United Kingdom and place responsibility for further actions with other nations.	**Energy demand** Editorial argues energy source is needed specifically to meet energy needs in the United Kingdom.
Push non-transformative solutions	**Illegitimate Advocates** Advocates for action are delegitimised, either explicitly or implicitly deemed untrustworthy or having ulterior motives for their advocacy.	**Energy demand** Editorial argues energy source is needed specifically to meet energy needs in the United Kingdom. **Economic Benefits** Editorial argues that energy sources are economically beneficial to the United Kingdom and/or global economy **Illegitimate supporters/opposers** Editorial delegitimises and dismisses supporters or opposers of an energy source or argues they have alternative motives for supporting it.
Emphasise the downsides of climate policies	**Economic Cost** Editorial presents climate action as too expensive to justify	**Economic cost** Editorial argues energy source is too expensive to justify use
Surrender to climate change	**Opportunity** Editorial presents climate change as having positive impacts, and therefore no action is required.	

In what follows, we present key examples from the quantitative and qualitative analysis to give insight into both the trends in these narratives over time across the right and left-leaning media and the nature of these discourses. In this, we give particular emphasis to examples that highlight the subtlety of discourses of delay compared with more overt climate sceptical narratives. We argue this subtlety brings with it a need within analysis for attentiveness to the sentiment of climate discourses, with narratives that may at first appear as advocating for climate change action also embedding delay discourses. For each of the codes that we have identified as corresponding with Lamb et al.’s delay typology, we look at the quantitative trends and the qualitative data examples to show how these types of discourse manifest.

First, our ‘fairness’ code speaks to Lamb et al.’s (2020) ‘Redirect Responsibility’ discourse through their concepts of ‘whataboutism’ (the idea that the UK’s greenhouse gas emissions are smaller than those of other countries, so the responsibility to reduce emissions does not lie with the United Kingdom) and ‘the free rider excuse’ (reducing emissions negatively impacts the United Kingdom, other countries will take advantage of this). For example: ‘The country mostly responsible for carbon emissions is not Britain, indeed it is a leader in cutting them [. . .] If anything the protesters should be demonstrating outside the Chinese and US embassies, not blocking London’s streets’ (The Times, 16/04/2019, *climate dataset*).

The ‘fairness’ code features mostly in right-leaning editorials (being present in 16% of all right-leaning editorials, compared to 0% of left-leaning editorials and only 1% of centre-leaning editorials) and exhibits an upwards trend over time, with a spike in 2016 and again in 2019 (see Figure 4 in Supplemental Information), the latter coinciding with the period when outright climate scepticism was last identifiable in the data set. This delay discourse is also identified through our ‘energy demand’ code in the energy dataset. Here, the analysis is complicated because this code spans two discourses of delay, with the main narrative primarily relating to non-transformative solutions discourse (discussed next). However, there are also connections to fairness narratives as editorials insisted the UK’s energy demand concerns were more important than addressing climate change, which was positioned as benefitting ‘others’. This then related closely to the fairness code and Lamb et al.’s corresponding delay discourse as such narratives also redirect responsibility albeit in a less overt way, by foregrounding narratives that suggest others stand to benefit from addressing climate change more than the United Kingdom. This subverts analysis that aims to galvanise action by highlighting how different places have varying vulnerability to climate change by translating it into a delay narrative that characterises UK action as non-beneficial to national interests.

This takes us to the second, and most dominant, delay narrative within our dataset, that of ‘Pushing Non-transformative Solutions’. The codes corresponding with this delay discourse again arise across the climate change and energy datasets and take different forms. The most clear-cut way in which this discourse is evident in our data is, however, in a wider analysis of the overall stance adopted within editorials to the different transformative (renewable energy technologies) and non-transformative (gas fracking) solutions we analysed within our dataset. Though overall the data showed support increasing from 28% of pro-renewable editorials in 2011 to 57% of pro-renewable editorials in 2021 and a decline in anti-renewable editorials, the right-wing editorials overwhelming positioned themselves firmly against renewables. For the right-wing energy editorials, 92% were anti-renewables, while 89% were pro-fracking.

The most common justifications for pro-fracking and anti-renewables positions were found within our ‘energy demand’ and ‘economic benefit’ themes, wherein articles argued that fracking was required to meet the UK’s energy needs and would have a positive impact on the economy, as compared to renewables, which have a negative economic cost (i.e. too expensive) and as not providing sufficient energy to meet the UK’s needs. For example, one editorial read:

‘Britain has virtually ceased coal production and its belated efforts to revive a nuclear programme are now hampered by the deep freeze into which relations with China have been plunged. The country’s future is being gambled on the success of renewables, with offshore wind farms the size of Yorkshire required to produce enough electricity, an approach that hinges on the vagaries of the weather’ (The Telegraph, 20/09/2021, *energy dataset*).

This demonstrates the idea that renewable energy is unreliable, necessitating other more ‘traditional’ sources of power, in this case coal and nuclear.

These more overt anti-stances on renewables and pro-stances on non-transformative options for power were set amid subtler narratives articulated within our codes for ‘illegitimate supporters/opposition/advocates’ across our energy and climate datasets, which also aligns with Lamb et al.’s ‘Push Non-transformative Solutions’ delay discourse. These codes refer to stories focused on the delegitimisation of certain actors who call for greater climate action (e.g. activists, policy-makers, global leaders). This more subtle narrative about specific voices in climate change debates served to delegitimise both calls for more urgent and substantial action on climate change, and voices opposing non-transformative solutions. The proportion of editorials including the code ‘illegitimate support’ in the climate dataset rose from 2% in 2018 to 22% in 2019, making it the most common theme justifying climate inaction in 2019. Nearly 40% of all editorials containing the ‘illegitimate support’ theme were published in 2019 alone. The prevalence of this theme in particular can likely be ascribed, at least in part, to the rise of Extinction Rebellion and the Fridays For Future school strikes in 2019.

Editorials relied on ‘metaphors and catch-phrases’ (Gavin and Marshall, 2011: 1036) to construct a specific discourse surrounding these protest movements in the (almost exclusively) right-leaning publications. For example, words such as ‘cult’, ‘pious’ and ‘extremist’ are common. Specific criticisms of the protesters vary from disagreeing with their tactics: ‘The group’s cause may be well-intentioned but its methods have tested people’s patience’ (The Times, 18/10/2019, *climate dataset*), to dismiss the whole group as ‘environmental extremists, anti-capitalists and poundshop anarchists’ (The Daily Mail, 07/09/2020, *climate dataset*). Within the energy data set, it was striking to find that the ‘illegitimate opposition’ discourse arose in relation to gas fracking but not nuclear or renewables – meaning it was reserved for those who opposed fossil fuels. Nearly a fifth (19%) of all editorials that were about fracking contained the ‘illegitimate opposition’ code.

Also common in the editorials across both datasets were themes which relate to the third delay discourse ‘Downsides of Climate Politics’. Our theme ‘economic cost’ of climate action, and of renewable energy options, demonstrates this most clearly. The ‘economic costs’ theme was the most common theme used to oppose climate action in the climate dataset, emphasising the delay argument that climate action was too costly to justify. For example, this quote from The Sun article in 2021 links the notion of ‘financial pain’ with political costs by invoking the idea of the need to ‘take voters with you’.

**Economic Cost**: ‘That his (Prime Minister) arbitrary deadlines and grand announcements will simply come true? In fact they threaten acute financial pain for millions. The Sun is campaigning for a greener planet. But it’s all very well Boris declaring, before the G7 summit, that Cornwall will be the first county to hit zero emissions. Have its people been told what that actually means? Unaffordable battery cars, capable only of short trips. Punitive gas bills. Their reliable central heating stripped out and replaced at their expense. Do not just impose all this on Britain, PM. You must take voters with you. And if you make them poorer, expect flak’ (The Sun, 08/06/2021 – *energy dataset*).

Most striking in the above quote is the insertion of the phrase ‘The Sun is campaigning for a greener planet. But. . .’ This is particularly evocative in expressing the ways that discourses of delay operate, with far subtler and less overt articulation of ideas and messages that undermine public and political support for the urgent actions that are required for climate change.

The last of Lamb et al.’s discourses of delay ‘Surrender to Climate Change’, which refers to messages that ‘raise doubt that mitigation is (still) possible, pointing to seemingly insurmountable political, social or biophysical challenges’ (Lamb et al., 2020: 4) was less evident than the others within our analysis. For example, this discourse is most closely related to our ‘Opportunity 1’ theme, which presented climate change as having positive impacts for the United Kingdom, therefore not requiring action to mitigate it. This is of course not directly aligned with Lamb et al.’s characterisation of this discourse but could nevertheless be viewed as a surrender narrative, albeit one that is couched in the idea that there is no need to act, rather that it being insurmountable. ‘Opportunity 1’ was present in less than 1% of the editorials in the climate dataset.

In 2019, our codes that align with the delay discourses of ‘Redirecting Responsibility’, ‘Non-transformative Solutions’ and ‘Downsides of Climate Politics/Action’, saw a significant spike compared to previous years (see Figure 4 in Supplemental Information). This suggests that while the scepticism and denial of climate change science has faded away in UK editorials over the period 2011–2021, other reasons to justify inaction have increased. There is perhaps reason to believe that some of the issues outlined here (economic cost of climate action, fairness) may have become more pertinent since the end of the current study as a result of the global energy crisis and Russia’s invasion of Ukraine. Ultimately, this analysis is suggestive of a new battle ground for climate change action, no longer around whether action itself is imperative but what form it should take and how quickly it should be enacted. This more insidious form of climate scepticism is mixed in with overt messages about ‘campaigning for a greener future’ or calls for climate action, while simultaneously undermining solutions that are key to achieving it.

## 5. Concluding discussion

In response to our research questions, we found (1) that UK newspaper editorials substantially more often represented a pro-climate action stance, than an anti-climate action stance, over the entire period of 2011–2021, with an upwards trend over the period of pro-climate action sentiment. (2) We also found evidence of multiple discourses of delay narratives present in UK newspaper editorials; for example, often the ‘Pushing Non-Transformative Solutions’ discourse was present in editorials arguing, for example, that fracking was a more suitable energy supply for the United Kingdom than renewable wind turbines. Similarly, we found evidence of the ‘Redirect Responsibility’ discourse in editorials which used ‘whataboutism’ arguments pointing blame at nations other than the United Kingdom for carbon emissions. (3) We found that right-wing sources were by far more likely to publish narratives and themes in line with delay discourses than left-wing sources.

The results highlight the overwhelming shift away from overt climate change scepticism and opposition to climate action, most notably in the right-leaning newspapers, but also the presence of more complex climate sceptical messaging used by newspaper editorials, providing crucial evidence for ‘discourses of delay’ (Lamb et al., 2020). This finding was supported by an analysis of a dataset that spans editorials which discuss both climate change and energy supply (renewables, nuclear and gas fracking), and which spans across left-leaning and right-leaning UK news media publications. With editorials generally under-researched but representing a key form of agenda-setting publication with political and policy influence, this analysis offers insight and evidence for the ways that climate change scepticism is evolving and developing within public discourse.

It is clear from this analysis that there has been a huge decrease in UK newspaper editorials displaying overt climate change scepticism articulated through narratives such as uncertainty surrounding science. There has also been a significant increase in those editorials supporting climate action, even (and especially) among right-leaning publications, which historically have opposed climate action. This perhaps can be attributed to multiple political and economic factors, including the Conservative government’s support of climate action and its commitment to a net-zero by 2050 target. Public awareness of the need for, and support of, climate action has also seen an increase over the decade 2011–2021 partly due to the mounting threat of climate change as charted by multiple IPCC reports over the timeframe, major international events such as the Paris Agreement and COP26 in the United Kingdom, and global protest movements such as Fridays For Future and Extinction Rebellion having a strong presence in the United Kingdom.

However, and crucially, our findings show that many editorials still contain narratives that push back against the need for climate action, but in a different way to those historically observed anti-climate action or sceptic discourses. Traditional Type 1 scepticism (denial of the existence of a warming trend) has essentially disappeared from UK editorials, despite past research showing UK media contains a large volume of climate change scepticism compared to other countries (Painter and Ashe, 2012). Yet a more insidious form of scepticism, which can be characterised in terms of ‘discourses of delay’ (Lamb et al., 2020), has prevailed and even increased in the same time period. This study therefore provides empirical support for Lamb et al.’s (2020) discourses of delay concept and speaks to the emerging literature on the significance of delay discourses across various areas of UK public and political debate (Cherry et al., 2024; Nisbett et al., 2024).

Right-leaning newspapers in particular continue to use themes that justify calls for reducing climate action, even if no longer explicitly arguing that action is not needed or justified by the science. For example, it is common for editorials to suggest responsibility for climate action does not lie with the United Kingdom but with other nations with high emissions, notably China, or to convey anti-renewables narratives while advancing pro-fracking (fossil fuel) discourses. Even more subtly, they frequently criticise climate activist groups such as Extinction Rebellion, who call for more urgent action, dismissing these groups (and their arguments) as extremist. They mount stories of economic costs of action together with tales of political costs emphasising the downsides of climate politics even amid narratives framed as supportive of climate action. Together, these themes create narratives which can be captured by the label of ‘discourses of delay’ (Lamb et al., 2020) and convey a new wave of climate scepticism, one which runs through stories not only directly connected to climate change but more broadly related to the set of changes required to address it, such as within the energy-related stories analysed here.

Despite recent debates over digital journalism, UK mainstream newspapers are considered to retain a strong agenda-setting power in society (Painter, 2011), and analysis of the coverage of climate and energy supply issues in these newspapers remains important. Opinion journalism, in particular, including editorials, is especially useful to understand the prevailing perspectives and voices of the powerful in society. While this study provides a novel addition to the literature on media representations of climate scepticism, offering a longitudinal analysis of UK opinion journalism, there are areas of future research which could usefully build on these findings. For example, a similar analysis of UK media beyond newspapers would be beneficial. While the agenda-setting power of newspapers makes them significant, it is also important to understand TV media (a consistently more popular site of news for the public than print media) and social media (rapidly increasing in significance for news, particularly among younger demographics), to investigate whether results hold across multiple media. Further, future research could more carefully consider the relationship between climate change and energy supply issues in UK media, as the treatment of the two datasets as separate in this study may have prevented certain connections or nuances from being investigated.

This analysis has implications for thinking about how to advance and renew climate change communication. Given the increasingly contested debates around Net Zero in UK policy and other related climate/energy issues (Paterson et al., 2023), it is important to understand how discourses alternative to denial can propagate and spread through media narratives, for example, through the ‘delay’ discourses discussed here. The public looks to the media for knowledge and understanding of climate change (Schäfer and Schlichting, 2014), and discourses of delay, which contradict scientific consensus on action, create a barrier to this understanding. The discourses of delay create doubt in the minds of readers, not over the existence of warming as in the past (Gavin and Marshall, 2011), but over the most effective solutions and actions to reduce emissions and the speed at which they can and should be enacted, creating a barrier to greater public engagement with climate change and action. As sceptical discourse shifts from the overt denial of scientific evidence to the more subtle delay narratives shown here, perhaps the ways in which those seeking to advance action on climate change communicate needs to change too. In the analysis here, we found that the ‘threat’ code wherein the dire consequences of the impacts of climate change are articulated was the most common by some distance in the climate dataset, and yet was the least used theme by the right-leaning media, suggesting that some typically left-leaning narratives are focused more strongly on communicating the threat of climate change. This may mean that while the right-leaning press is focused on stories that undermine and delay action, the left-leaning press is not similarly focused on stories of proactive or positive action. Grounding these threat narratives within articles about how people can and do live differently and thrive in a world that is also addressing the threat of climate change is a potential route forward for communicating about this complex issue. As climate change and energy issues become an increasingly polarising topic of public, political and media debate in the United Kingdom, the relative prominence of these discourses of delay should be carefully considered with regard to the accuracy of information about government policies for climate action.

## Supplemental Material

sj-pdf-1-pus-10.1177_09636625251315446 – Supplemental material for From climate scepticism to discourses of delay in UK editorials Supplemental material, sj-pdf-1-pus-10.1177_09636625251315446 for From climate scepticism to discourses of delay in UK editorials by Sylvia Hayes, Josh Gabbatiss and Catherine Butler in Public Understanding of Science
