# Supplementary material for: From climate scepticism to discourses of delay in UK editorials
Source: Public Underst Sci. 2025 Mar 11;34(7):832–51. doi: 10.1177/09636625251315446 (PMC12449614; doi:10.1177/09636625251315446)
Supplement: sj-pdf-1-pus-10.1177_09636625251315446 – Supplemental material for From climate scepticism to discourses of delay in UK editorials [file sj-pdf-1-pus-10.1177_09636625251315446.pdf]

SUPPLEMENTAL INFORMATION: From Climate Scepticism to Discourses of Delay in UK Editorials

SYLVIA HAYES[1*], JOSH GABBATISS[2], CATHERINE BUTLER[1]

[1]Geography, University of Exeter, Amory Building, Rennes Drive, Exeter, EX4 4RJ, UK. *Corresponding author: sh737@exeter.ac.uk [2]Carbon Brief, UK

SUPPLEMENTAL INFORMATION CONTENTS:

1: Figure: "Right-leaning editorials: themes associated with "no action" over time" ______ p.2

2: Codebooks ______ p.3

3: Inter-coder reliability information ______ p.16

SUPPLEMENTAL INFORMATION 1: Figure: "Right-leaning editorials: themes associated with "no action" over time"

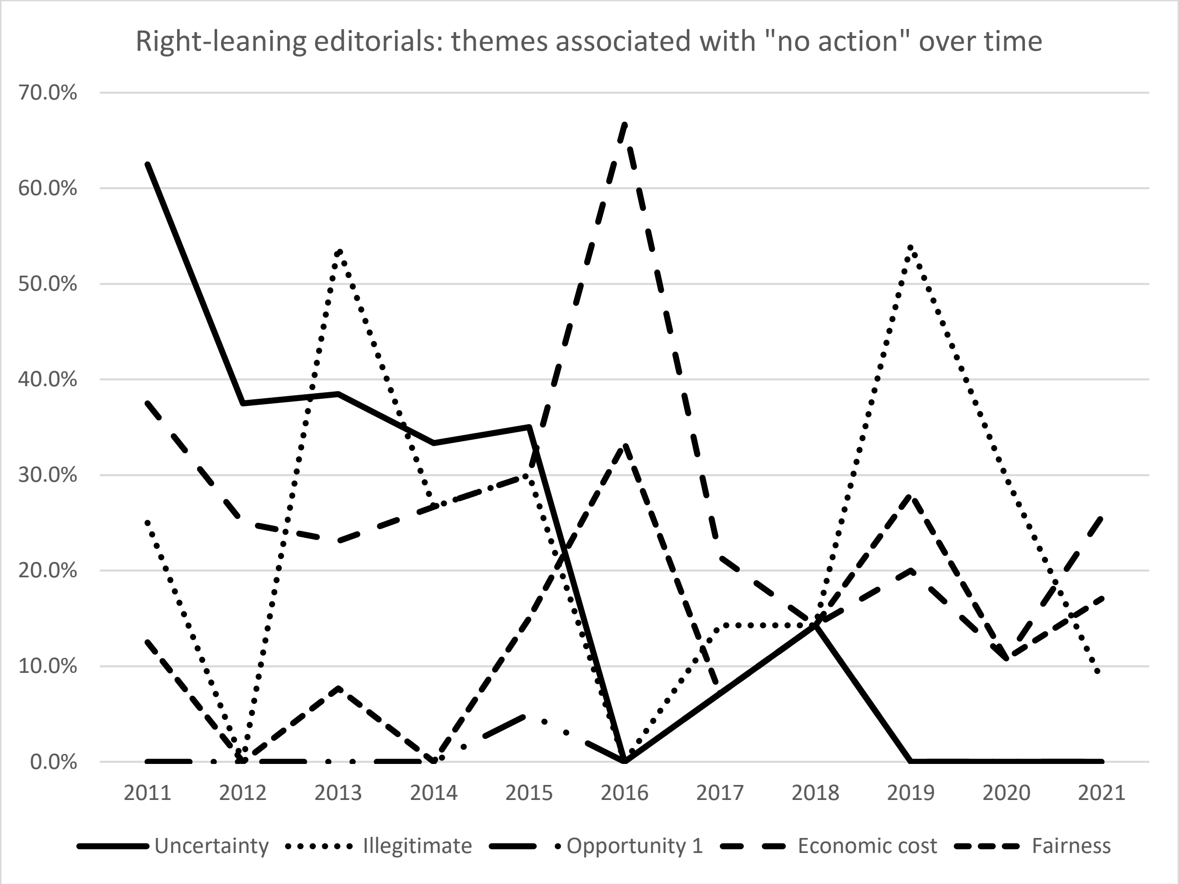

Figure: Percentage of editorials each year which contain each theme associated with "no climate action" in right-leaning editorials

SUPPLEMENTAL INFORMATION 2: CODEBOOKS

1. Creating the datasets

The primary focus of the analysis is climate change editorials, so all editorials which contain the keywords “climate change” or “climate action” or “global warming” or “global heating” or “greenhouse gas emissions” or “Paris Agreement” or “Paris climate deal” or “COP26” make up the *climate change* dataset.

See section 3 for creation of *energy* dataset.

For each of *climate* and *energy* datasets, all editorials are read in full, and either deemed not relevant (according to the descriptions below) or coded (according to relevant coding schema).

2. Climate Change dataset

2.1. DEVELOPING THE CODING SCHEMA

The coding schema was developed reflexively using a two-step process of both inductive and deductive methods. First, a literature review was conducted of similar media analyses on climate and energy issues (see for example O’Neill et al., 2015; Painter, 2011; Culley et al., 2010; Djerf-Pierre et al., 2015; Rochyadi-Reetz et al., 2019) to create a provisional coding scheme. This provisional coding scheme was then tested and reflexively refined to better suit the data on a 10% sample of the data by two coders (SH and JG).

2.2. INSTRUCTIONS FOR CODERS

2.2.1. Determining relevance

If an editorial does not discuss the *issue* of climate change, editorial is deemed irrelevant for this analysis and left blank in dataset. For instance, if editorial only contains the keyword “climate change” in the context of “Secretary of State for Energy and Climate Change”, and there are no other mentions of keyword(s), editorial is not considered relevant.

Editorials in the *Climate Change* dataset are then coded according to a two-step coding schema, below.

2.2.2. Meta-frames

First, code for the overarching “meta-frame” of the editorial, referring to the overarching stance of the editorial in full. Each editorial can only be coded for one meta-frame.

To be coded as either more action or less/no action, editorial *must have a stance (explicit or implicit) on climate change action*; that is, action to mitigate against the impacts of climate change, and/or to reduce carbon emissions to limit future warming. *Calling for increased flood defences (or similar) alone is not sufficient to warrant coding for “more action”. Editorial must call for action on climate change, not on other issues.*

**More action required**	Editorial calls for more policies/action to reduce the effects of climate change. Editorial may praise the work currently being done in the UK/globally, e.g. praising specific policies which aim to reduce the effects of climate change, but must also argue that current action is insufficient to the scale of the problem<br><br>NOTE: code for action *in the UK*. If editorial doesn’t discuss specific countries, use this code for global action

**Less or no action required**	Editorial calls for either *no* action to mitigate the effects of climate change, OR calls for *less* action than is currently being done.<br><br>Editorial will *either* contain a “type 1 or 2 climate sceptic” viewpoint (Painter, 2011), where the scientific consensus of anthropogenic climate change is questioned or undermined, *OR* will argue that the actions that the UK/the world is currently taking (at time of editorial) are sufficient or more than sufficient to reduce the harmful effects of climate change, negating the need for further action than what is currently being done.<br><br>Editorials are likely to refer to negative impacts of climate action policies, particularly on “working people” or “ordinary people”<br><br>NOTE: code for action *in the UK*. E.g. if editorial argues for the need for action, but states that the UK already does too much/enough, then use this code. If editorial doesn’t discuss specific countries, use this code for global action
**Mixed/unclear**	Editorial is purely informational (e.g. explaining the science of climate change or explaining a specific event without taking a position on whether action should be taken or not), OR argues contradictory points.<br><br>For example, editorial may argue in favour of some policies to mitigate climate action, but argue against others<br><br>For example, editorials are likely to refer to the need for policies to help reduce individual climate emissions by flying less and eating less meat, but also argue against the need for overall zero or net-zero emissions.<br><br>Note: this does not refer to editorials which argue against policies on the basis that *current policies are sufficient*. if this is the case, code as “less action”<br><br>NOTE: the presence of “the other side” of the argument alone does not represent a mixed meta-frame. An editorial may only be coded for mixed if “both sides” are left unchallenged.<br><br>NOTE: use this code for UNCLEAR/UNDETERMINED editorials also

2.2.3 Thematic codes

Second, code for “thematic codes” present in editorial. These codes aim to capture the nuance of arguments present in editorials. Each editorial can contain multiple and/or contradictory codes. Code for every element present in editorial *which represents the stance of the editorial* - do not code for arguments which are discredited by the editorial. E.g. if editorial discusses the existence of “climate deniers” who do not accept the scientific consensus, but the editorial itself clearly states that there *is* sufficient scientific consensus, then do not code for uncertainty.

These codes relate to a specific meta-frame, but are coded *independently* of meta-frame. For instance, an editorial can be coded for the “more action” meta-frame, but can also contain one or more thematic codes which relate to the “less action” meta-frame.

This allows the analysis to capture contradictions which were found to be present in some editorials during sampling.

See table below for details.

Theme	Description	Related meta-frame	Origin	Common storylines/example phrases
**Uncertainty of Science**	There remains scientific uncertainty over EITHER the existence of climate change, OR the cause. For instance, editorial may state that climate change is happening, but is not caused by humans. Editorial casts doubt on the scientific evidence of anthropogenic climate change.<br><br>NOTE: if editorial mentions but dismisses/quashes the uncertainty, or discussed uncertainty in a scientific way that still acknowledges consensus, code for Settled Science instead<br><br>NOTE: this code refers to the *scientific evidence* for/against climate change, not *scientists* as personalities. If editorial delegitimises/dismisses specific scientists, or assumes dubious motives of scientists (e.g. as a result of the Climategate emails), code for Illegitimate advocates	Less action	Adapted from O'Neill et al 2015, inductive	• "huge doubts" surrounding the science of climate change<br>• "[Climate] science is disputed, some of it discredited"<br>• "More recently, some forecasters have claimed that the climate may actually get a little colder over the next few decades as a consequence of reduced solar flare activity"
**Illegitimate advocates**	Advocates for action are delegitimised, either explicitly or implicitly deemed untrustworthy or having ulterior motives for their advocacy. "Advocates" includes anyone calling for action e.g. scientists, politicians, public figures (e.g. celebrities), activists.<br><br>NOTE: editorial may welcome the overall message of the advocates (that action is needed), while still undermining the specific	Less action	Inductive	• Often applied to protest groups e.g. Extinction Rebellion. They are right that action is needed, but Britain already leads Europe/the world in emissions reduction, and activists are dismissed as "hippies" etc.<br>• Commonly used language to undermine advocates includes "green lobby", "alarmists", "doomsayers"<br>• Climate protesters (e.g. school strikers) are "infantile", "misguided"

	groups/personalities delivering the message.<br><br>NOTE: this code includes reference to the Climategate emails, where this is used to discredit personalities			• “There is no doubt that the issue of climate change now has great salience and that Extinction Rebellion is due some of the credit. Yet the protesters seem strangely lacking in anything more to say beyond their stock-in-trade of rampant exaggeration.”
**Opportunity 1: benefits of global heating**	If the climate is changing (which may be uncertain), there are benefits to this e.g. warmer weather. Negative impacts may be dismissed or ignored.<br>Editorial presents climate change as having positive impacts, and therefore no action is required.<br><br>NOTE: if opportunities are purely economic (e.g. “green jobs”) code for Economic benefits of action	Less action	O’Neill et al 2015	• Warmer summers<br>• Increased transport access around e.g. Arctic as a result of melting sea ice
**Economic cost of action**	Economic justifications for no action/reduced action. Likely to refer to economic growth.<br>Editorial presents climate action as too expensive to justify.	Less action	Adapted from O’Neill et al 2015, inductive	• Action is “costly” to the British taxpayer<br>• Action is “damaging” to economic growth
**Fairness/responsibility**	A UK-specific code. Editorial uses notions of fairness/responsibility to justify a call for no action or reduced action in the UK. Likely to refer to action already being taken by UK, and place responsibility for further actions with other nations.<br>Editorial must compare UK to other nations to be coded here.	Less action	Inductive	• Relates to the “free rider” argument<br>• Likely to refer to (lack of) climate action from China<br>• "this government already leads the civilised world in carbon reduction"
**Settled Science**	There is broad scientific consensus, and/or considerable scientific evidence of the need for action. Any discussion of scientific uncertainty is dismissed or quashed (if uncertainty is left unchallenged, code for	More action	O’Neill et al 2015	• The debate is over; science has spoken, and others must now act<br>• “climate change is real”<br>• “Work by hundreds of scientists in dozens of countries confirms a 95% probability that carbon

	uncertainty of science). Editorial argues that the debate over climate change is over.			emissions account for at least half of the observed increase in global warming"<br>• "contention is dangerously false"<br>• [discussing extreme weather] "It seems almost certain that man-made climate change has a role in such events"
**Threat**	Impacts of climate change (either existing or future) are dire, with severe consequences. Climate change represents a threat to human and/or animal populations.<br>Editorial must explicitly state the *scale* of the problem, for example referring to the issue as "catastrophic" or "existential threat"<br>May refer to biodiversity loss. May refer to extreme weather events.<br>NOTE: for extreme weather events, a clear link to climate change must be made.	More action	Adapted from O'Neill et al 2015	• "catastrophic" climate change impacts<br>• Any discussion of *negative* impacts of climate change<br>• Can refer to impacts in UK or worldwide, now or in the future<br>• "2019 was the second-hottest year on record" *with link to climate change* – any mention of "record temperatures" or similar
**Economic benefit of action**	The economic costs of action may be high, but the cost of NOT acting to mitigate the effects of climate change will be higher, creating a strong economic case for action now. May also refer to outright economic benefits such as "green jobs" or "green economy"	More action	Adapted from O'Neill et al 2015, inductive	• Likely to refer to "green jobs", "green new deal" or "green economy" etc.<br>• "cutting emissions will not be cost-free [...] But persisting in the mistakes of the present decade for another 10 years would [...] be the greatest foolishness of all."<br>• "The coronavirus-induced recession is widely accepted as an opportunity to reset and rebuild the economy to take the environmental challenge seriously."
**Opportunity 2: social benefits of action**	Climate change has provided us with an opportunity to reimagine how we live. There are additional benefits to action. May refer to happier, healthier communities as a knock-on effect of action.	More action	Adapted from O'Neill et al 2015, inductive	• Reduced air pollution as a side effect of mitigating climate change<br>• "the most important and hopeful revolution in technology since the smoke began spewing out of British chimneys,"

	NOTE: if benefits of action are strictly economic (e.g. "green jobs"), code for Economic benefit			
**Morality/ethics**	There is a strong moral/ethical justification for action *on climate change*. Editorial argues we (in the UK or globally) have a *responsibility* for more action. May include religious calls to action. Includes explicit links between climate change and poverty, development, or intergenerational (in)justice.<br><br>NOTE: must be moral call for action on *climate change* explicitly. E.g. if editorial discusses victims of flooding as needing help, but does *not* explicitly link to climate change and the moral justification for *action on climate change*, do not code.	More action	Adapted from O'Neill et al 2015, inductive	• May refer to need to act on behalf of / for benefit of "future generations"<br>• Can be EITHER UK specific: as a wealthy nation, we have a moral obligation/responsibility to act to help those who are vulnerable in the world: "the UK — a minor contributor to global emissions and other forms of pollution — can achieve most by pressing for international action, supporting developing countries and making its domestic policy an example for others to follow"<br>• OR global: e.g. notions of collective humanity coming together to solve problem, or issues of development/justice meaning the poor/vulnerable are most at risk and therefore "we" (global community) must act
**Political/ideological struggle**	Climate change is a political issue, involving arguments/conflicts between politicians. Editorial may argue a particular side as "correct", or may present balanced arguments on both sides.	Balanced	O'Neill et al 2015	• May refer to battle of power between nations or political groups<br>• Includes any reference to policies aiming to address the issue of climate change

3. Energy dataset: instructions for coders

The energy dataset is made up of editorials which contain one or more of the following keywords:

wind power or wind farm or wind energy or renewable or nuclear power or nuclear energy or fracking or shale gas or renewable energy or renewable power or solar power or solar energy or solar or fracking or hydraulic fracturing

3.1 Determining Relevance

To be considered relevant, editorials must discuss one or more of: nuclear power, fracking, or renewable energies. This was determined from sampling, where it was clear that these are the most common energy issues appearing in the dataset.

Therefore, any editorial in this dataset which does not mention one of these three energy sources is considered not relevant for coding. For example, editorials which are about the high cost of energy, but do not mention any of nuclear power, fracking, or renewable energies, are considered not relevant for coding.

For the purposes of this study, “renewables” refers to only: wind power, solar power, and hydro-power. We acknowledge there are other forms of renewable energy, but for the purposes of this study we focus only on these key energies which appear most commonly in the dataset.

2.1 Developing the coding schema

The coding schema was developed reflexively, using a mixture of inductive and deductive methods. A literature review of media analyses of different forms of energy was conducted, and helped inspire early versions of the coding schema which were then refined and tested against a sample from the dataset. (See e.g. Culley et al., 2010; Delshad & Raymon, 2013; Djerf-Pierre et al., 2015; Rochyadi-Reetz et al., 2019)

2.2 Meta-frames

First, code for the overarching “meta-frame” of the editorial, referring to the stance that the editorial takes on one or more of the energy sources being investigated. Each editorial can be multiple coded if appropriate. For example, an editorial may state that renewable energy sources are needed in the UK, because fracking is too costly and damaging to the local environment. In this case, the editorial would be coded for “Pro-renewables” *and* “Anti-fracking”.

These meta-frames refer to the overall argument being made by the editorial in full. If, for instance, an editorial discusses the disadvantages of fracking only to conclude that the benefits outweigh the costs, then the editorial should be coded for “Pro-fracking”. Often, the final line/sentence(s) of an editorial are most revealing as to the overall stance of the editorial.

See table below for details.

**Nuclear**	Pro-nuclear energy	Editorial overall supports of the use of nuclear energy in the UK (or worldwide)
	Anti-nuclear energy	Editorial overall opposes to the use of nuclear energy in the UK (or worldwide)
	Mixed/unclear	Editorial either argues both for and against, without taking an overt stance for or against use of nuclear power in the UK (or

		worldwide), or is purely providing information on the use of nuclear power
**Fracking**	Pro-fracking	Editorial overall supports of the use of hydraulic fracking in the UK (or worldwide)
	Anti-fracking	Editorial overall opposes to the use of hydraulic fracking in the UK (or worldwide)
	Mixed/unclear	Editorial either argues both for and against, without taking an overt stance for or against the use of hydraulic fracking in the UK (or worldwide), or is purely providing information on the use of hydraulic fracking
**Renewables**	Pro-renewables	Editorial overall supports of the use of one or more of: wind power, solar power, or hydro-power in the UK (or worldwide)
	Anti-renewables	Editorial overall opposes to the use of one or more of: wind power, solar power, or hydro-power in the UK (or worldwide)
	Mixed/unclear	Editorial either argues both for and against, without taking an overt stance for or against the use of renewable energy in the UK (or worldwide), or is purely providing information on the use of renewable energy

2.3 Thematic codes

Second, code for "thematic codes" present in editorial. These codes aim to capture the nuance of arguments present in editorials. Each editorial can contain multiple and/or contradictory codes. Code for every element present in editorial *which represents the stance of the editorial*. For instance, do not code for arguments which are discredited by the editorial. For instance: "There are also fears about tremors, fuelled by two mini-earthquakes near Blackpool. Although there is as yet no evidence that seismic activity is increased by fracking". In this exert, do not code for "local environment costs" of earth tremors, as the risk is dismissed by the editorial.

Codes relate to meta-frame(s), but are coded *independently* of meta-frame(s). This allows the analysis to capture contradictions which were found to be present in some editorials during sampling.

The table below is applicable to each of: nuclear power, fracking, renewable energies.

Theme	Description	Origin	NUCLEAR Common storylines/examples	FRACKING Common storylines/examples	RENEWABLES common storylines/examples
**Economic costs**	Energy source is too expensive to justify use. Editorial may compare energy source to other (cheaper) sources. Editorial may dismiss energy source as “waste of money”, or argue that energy source won’t produce enough energy to be worth the cost to the taxpayer/government	Culley et al 2010<br>Delshad & Raymond, 2013	- Taxpayer money vs government subsidies, investment from foreign powers		- Wind turbines “don’t/won’t work” and are a waste of taxpayer money
**Climate (global environment) costs**	Energy source is damaging to global environment. This refers to either currently existing, or expected future damage. Editorial will likely relate energy source to climate change.<br><br>NOTE: if environmental damage is discussed only at local/community level, code for Local environment costs	Culley et al 2010<br>Delshad & Raymond, 2013,<br>Rochyadi-Reetz et al 2019	- Nuclear energy is cleaner than fossil fuels	- Continued reliance on fossil fuels	- Renewables produce no carbon emissions in energy production<br>- Assisting the UK reach climate/emission reduction goals
**Local environment/society costs**	Energy source is damaging to local area, EITHER local environment (e.g. damaging natural beauty of area, disturbing wildlife), OR socially damaging to local community (e.g. health and safety concerns of local residents). May refer to pollution of local area (if editorial discusses pollution of *global* environment e.g. carbon emissions, code for Climate costs)<br><br>NOTE: if costs are economic, code for Economic costs, even if	Culley et al 2010,<br>Delshad & Raymond, 2013,<br>Rochyadi-Reetz et al 2019		- Fracking causes earth tremors/earthquakes	- Wind turbines are “ugly”, “blight” on natural beauty of local area

	economic costs are relevant to local area				
**Illegitimate supporters**	Supporters of this energy source are delegitimised, dismissed, or implicitly or explicitly stated to have ulterior motives for their support. “Supporters” may refer to politicians, scientists, activists, public figures, or any other personality or group which outwardly supports the energy source.<br>May refer to “undeserving” corporations or foreign powers benefitting from the development/use of this energy source, rather than local area or UK benefitting.	Culley et al 2010	- Involvement of foreign powers e.g. China’s economic involvement in nuclear power plant is contrary to UK interest		- Editorial may dismiss “green lobby” or environmentalists
**Economic benefits**	Energy source is economically beneficial to the UK and/or the global economy.<br>This code includes discussion of boosting the UK economy, and/or notions of UK becoming a superpower/world-leader in this energy/technology.<br><br>NOTE: for UK energy independence, code for Energy Demand<br><br>NOTE: if editorial discusses economics only in terms of job creation *in local area*, code for local environment benefits. Job creation nation-wide falls under this code	Culley et al 2010, Delshad & Raymond, 2013			

**Energy Demand**	Energy source is needed specifically to meet energy demand in the UK. We are currently not meeting our energy needs, and this energy source will help boost UK energy production and "keep the lights on".<br>This code includes discussion of the need for UK energy independence; editorial may discuss current over-reliance on foreign powers for energy<br><br>NOTE: this code is only relevant for discussions of energy demand/UK energy mix. If editorial discusses UK becoming world-leader in a specific technology, code for Economic Benefits	Inductive			
**Climate (global environment) benefits**	Energy source provides a benefit to the global environment. Energy source is either an objective good for the global environment, or is beneficial *relative* to other existing energy sources.<br><br>Editorial will likely relate energy source to climate change – e.g. this energy source will reduce reliance on other, more CO2-emitting, sources<br><br>NOTE: if environmental benefits discussed only at local/community level (e.g. reduced pollution levels	Culley et al 2010, Delshad & Raymond, 2013, inductive	- Better than fossil fuels		- Renewable energy is necessary for meeting emissions reductions targets & fighting climate change

	in local area), code for Local environment benefits				
**Local environment/society benefits**	Energy source will bring environmental benefits to the local area (e.g. reduced pollution), or social benefits to the local community (e.g. job creation)<br><br>NOTE: this code refers *only* to benefits *in local area/to local residents*. Do not use this code for nationwide benefits	Culley et al 2010, Delshad & Raymond, 2013			
**Illegitimate opposition**	Editorial dismisses and delegitimises opposition personalities/groups, e.g. implicitly or explicitly deeming them untrustworthy or having ulterior motives. "Opposition" includes anyone speaking out against energy source, e.g. politicians, scientists, activists, public figures (e.g. celebrities)<br><br>Can refer to specific personalities, or opposition groups (e.g. activist groups) as a whole.<br><br>NOTE: editorial may welcome the overall message of the advocates (that climate change is a pressing issue), while still undermining the specific groups/personalities delivering the message.	Culley et al 2010, inductive		- "hysteria"<br>- Opposition is a "leafleting campaign by rent-a-mob"<br>- Editorial may agree with the overall message that we should move away from fossil fuels, but dismiss/delegitimise the activist(s) opposing fracking on these grounds	
**Political/ideological struggle**	Editorial presents the energy source as a political issue, involving	Inductive, O'Neill et al. 2015			

	arguments/conflicts between politicians.<br><br>Editorial may argue a particular side as “correct”, or may present balanced arguments on both sides.<br><br>NOTE: do not use this code for any mention of politicians. E.g. quoting UK Prime Minister alone does not justify this code. To be coded for PIS, editorial must present the energy source as an issue of politics involving disagreements between political/ideological groups/personalities<br><br>NOTE: this code does not capture whether the editorial is praising or criticising the politics involved, only captures whether the issue is discussed as an issue of politics				

SUPPLEMENTAL INFORMATION 3: Inter-coder reliability testing

Two coders, 10% sample of dataset coded independently.

All disagreements were discussed after this test until agreement was reached, and the codebook was then edited to reflect these changes. This explains the low ICR score for some variables, which were discussed after these scores and the codebook was updated to reflect the consensus which was reached through discussion. The inter-coder reliability score was treated as one approach among multiple to ensure the rigor of our analysis, and used it primarily as an initial point to prompt and guide discussion and debate among the authors regarding the coding, rather than using it as a single measure of the "success" of our codebook. See O'Connor and Joffe (2020) for more.

Average Krippendorff's Aplha score across all variables: 0.72.

**Variable/code**	**Scott's Pi**	**Cohen's Kappa**	**Krippendorff's Alpha (nominal)**	N Cases	N Decisions
More Action	89.50%	0.739	0.739	6	57
Less Action	100%	1	1	0	57
Balanced	94.70%	0.77	0.77	3	57
Uncertainty	100%	1	1	0	57
Illegitimate	94.70%	0.811	0.811	3	57
Opportunity 1	100%	1	1	0	57
Economic Cost	94.70%	0.698	0.701	3	57
Fairness	100%	1	1	0	57
Settled Science	93%	0.709	0.71	4	57
Threat	84.20%	0.674	0.674	9	57
Economic benefits	87.70%	0.619	0.623	7	57
Opportunity 2	94.70%	0.372	0.383	3	57
Morality/Ethics	78.90%	0.272	0.296	12	57
Political	73.70%	0.463	0.463	15	57
